# Differentiated kidney tubular cell-derived extracellular vesicles enhance maturation of tubuloids

**DOI:** 10.1186/s12951-022-01506-6

**Published:** 2022-07-15

**Authors:** Rafael Soares Lindoso, Fjodor A. Yousef Yengej, Franziska Voellmy, Maarten Altelaar, Estela Mancheño Juncosa, Theano Tsikari, Carola M. E. Ammerlaan, Bas W. M. Van Balkom, Maarten B. Rookmaaker, Marianne C. Verhaar, Rosalinde Masereeuw

**Affiliations:** 1 Division of Pharmacology, Utrecht Institute for Pharmaceutical Sciences, Utrecht University, Universiteitsweg 99, 3584 CG Utrecht, The Netherlands; 2 Institute of Biophysics Carlos Chagas Filho, Federal University of Rio de Janeiro, Rio de Janeiro, 21941-902 Brazil; 3 National Institute of Science and Technology for Regenerative Medicine-REGENERA, Federal University of Rio de Janeiro, Rio de Janeiro, 21941-902 Brazil; 4 Hubrecht Institute-Royal Netherlands Academy of Arts and Sciences, Utrecht, The Netherlands; 5 Department of Nephrology and Hypertension, University Medical Centre Utrecht, Utrecht, The Netherlands

**Keywords:** Extracellular vesicles, Kidney tubuloids, Maturation, Organic anion transporter 1, Proteomics, Bioengineered kidney tubules

## Abstract

The prevalence of end-stage kidney disease (ESKD) is rapidly increasing with the need for regenerative therapies. Adult stem cell derived kidney tubuloids have the potential to functionally mimic the adult kidney tubule, but still lack the expression of important transport proteins needed for waste removal. Here, we investigated the potential of extracellular vesicles (EVs) obtained from matured kidney tubular epithelial cells to modulate in vitro tubuloids functional maturation. We focused on organic anion transporter 1 (OAT1), one of the most important proteins involved in endogenous waste excretion. First, we show that EVs from engineered proximal tubule cells increased the expression of several transcription factors and epithelial transporters, resulting in improved OAT1 transport capacity. Next, a more in-depth proteomic data analysis showed that EVs can trigger various biological pathways, including mesenchymal-to-epithelial transition, which is crucial in the tubular epithelial maturation. Moreover, we demonstrated that the combination of EVs and tubuloid-derived cells can be used as part of a bioartificial kidney to generate a tight polarized epithelial monolayer with formation of dense cilia structures. In conclusion, EVs from kidney tubular epithelial cells can phenotypically improve in vitro tubuloid maturation, thereby enhancing their potential as functional units in regenerative or renal replacement therapies.

**Graphical Abstract:**

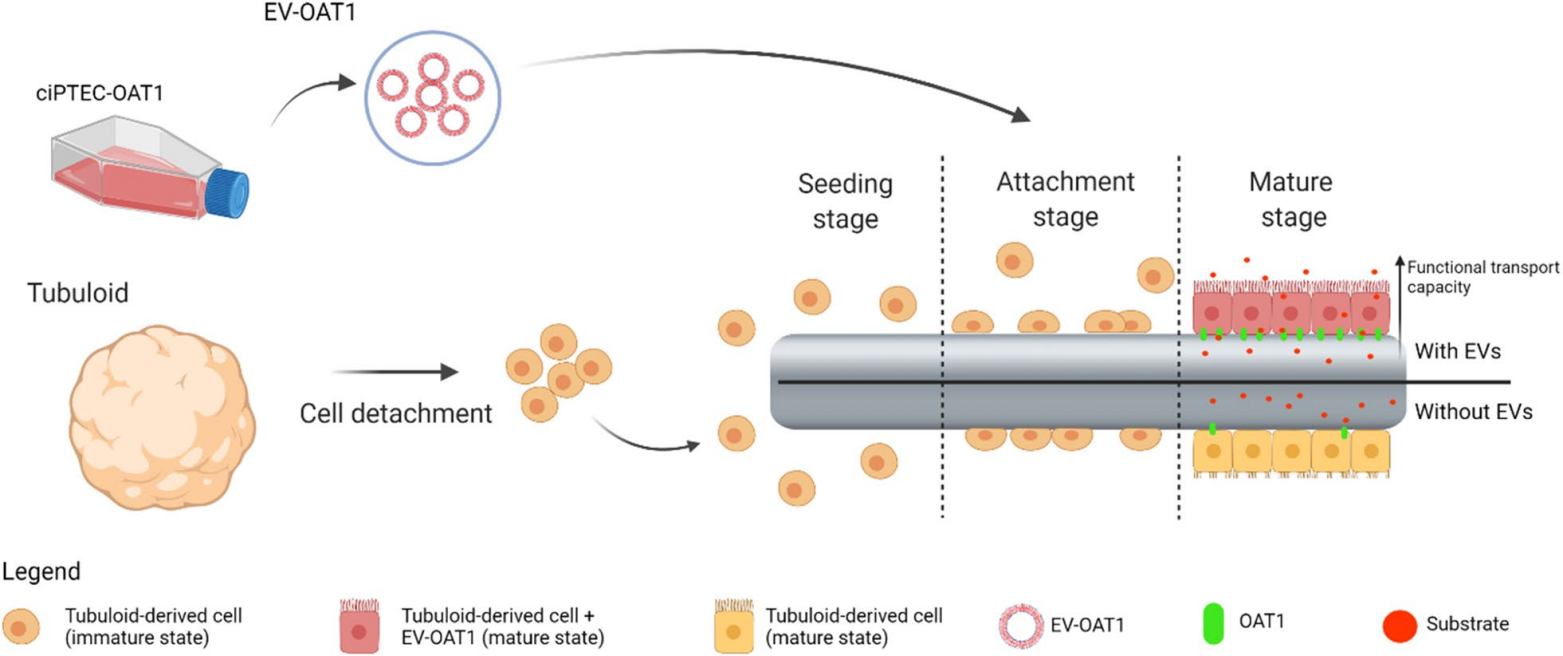

**Supplementary Information:**

The online version contains supplementary material available at 10.1186/s12951-022-01506-6.

## Translational statement

Tubuoids are three-dimensional multicellular structures that recapitulate kidney tubular function and hold great potential as cell source for bioartificial kidneys. However, the application of tubuloids into the clinic requires improvement of functional maturation. Herein, we demonstrate that EVs derived from tubular epithelial cells, overexpressing the epithelial transporter OAT1, are capable to improve tubuloid maturation by increasing the expression of different epithelial transporters and transcription factors that ultimately resulted in the improvement of cell polarization and transport capacity. Our findings show that EVs can be used as a tool to support the use of tubuloid in bioartificial kidneys, offering another strategy for their in vitro maturation. Understanding the mechanism involved in such EV mediated regulation mediated can support future therapeutic applications. Moreover, the use of EVs can be extended to other tissue models with several applications in drug screening, disease modeling and regenerative medicine.

## Introduction

End-stage kidney disease (ESKD) is a major health issue with increasingly high morbidity and mortality rates worldwide [1]. When kidney function declines below 10–15%, endogenous waste accumulates in blood and cause toxicity, which affects the functional capacity of the kidney and other organs [2]. The best treatment option is kidney transplantation, however, the shortage in donor availability and the high risk of transplant failure cause patients to rely on dialysis treatment. Yet, dialysis cannot fully substitute the spectrum of the kidney function [3]. In this context, new renal replacement treatment strategies must be developed such as the bioartificial kidney that can replicate the kidney’s metabolic and excretory function [4–7]. For clinical translation of such novel developments, it is critical to define a cell source that can represent the various specialized cell types present in the kidney and, at the same time, be biocompatible with the patient.

Organoids are three-dimensional structures derived from stem cells that reproduce in vitro the formation of near-physiological tissues [8]. Due to their self-renewal and self-organizational capabilities, organoids are state of the art cell culture models to study kidney development and disease [9]. Interestingly, progenitor cells derived from adult kidneys; upon culturing, allow for long-term expansion and generation of tubule-like structure, the tubuloids, with primary functional renal epithelium reflecting several aspects of all tubular regions of the mature nephron [10–12]. Despite being more mature than (induced) pluripotent stem cell-derived organoids, tubuloids do not yet present a fully developed phenotype, especially for proximal tubules and their capacity to promote transepithelial transport, limiting their application in biomedical, pharmacological and toxicological studies [13, 14].

The ability of the kidney to actively excrete metabolic waste, drugs and their metabolites is given by the presence of transporters in the membranes of proximal tubule cells [12]. Among these, organic anion transporter 1 (OAT1) is highly expressed that together with apical efflux pumps like multidrug resistance-associated proteins (MRPs) and breast cancer resistance protein (BCRP) contributes to the transfer of a large range of organic compounds from the blood circulation to the urine, in a highly controlled manner [15, 16]. As OAT1 is an important determinant of xenobiotic induced kidney injury [17], its absence limits the application of kidney-derived cells, organoids and tubuloids as screening platforms in the early phases of drug development but hampers also other applications such as in regenerative therapies. Thus, improving kidney cell maturation in vitro to reach expression of OAT1 and other proximal tubular transporters to levels close to native tissue is crucial for their predictive capacity as a preclinical test platform [18].

Strategies to improve kidney organoid and tubuloid maturation have focused on the use of different growth factors and aspects of the kidney microenvironment (e.g., 3D organization, vascularization, extracellular matrix, or fluid flow) [19–22]. In this context, the use of extracellular vesicles (EVs) has been described as a suitable strategy in regulating kidney regeneration [23, 24]. These vesicles are nanosized lipid bilayer structures that mediate cellular communication through the transfer of bioactive molecules, including proteins, nucleic acids and lipids [25]. Kidney EVs were shown to regulate cell fate and participate in intranephron communication in vivo by transferring unique proteins and RNAs that can modulate the functional activity of tubular epithelial cells, revealing a complex regulatory communication system [26–32].

As EVs can transfer the imprinting of originator cells to recipient cells, we investigated the potential of EVs derived from differentiated kidney proximal tubule cells overexpressing OAT1 to support tubuloid functional maturity by inducing OAT1 expression. Moreover, we investigated the mechanisms involved and explored the capacity of EVs to functionally mature tubuloids-based bioengineered tubules as functional units of a bioartificial kidney device.

## Material and methods

### Renal cell culture

Human conditionally immortalized proximal tubule epithelial cells that constitutively express organic anion transporter 1 (ciPTEC-OAT1; Cell4Pharma, Oss, The Netherlands) were used as source of EVs. Their generation was previously described [14]. In brief, the commercial vector containing OAT1 (pENTR201-hOAT1, Harvard Plasmids HsCD00044153) was transferred into a pLenti4/V5-DEST vector by LR recombinant reaction. Posteriorly, lentiviral stock containing OAT1 was produced by transfecting the pLenti expression constructs with packaging plasmid mix into the HEK293FT cell line. Finally, ciPTECs were exposed to lentiviral particles for 24 h and ciPTEC-OAT1 were selected and subcloned to obtain a homogeneous cell population. Expression levels were measured by qRT-PCR [14]. For culturing, the cells were maintained at 33 °C and 5% v/v CO_2_ to proliferate, until reaching up to 90% confluency, in DMEM/HAM’s F12 (Gibco), supplemented with 5 μg/ml insulin, 5 μg/ml transferrin, 5 μg/ml selenium, 35 ng/ml hydrocortisone, 10 ng/ml epidermal growth factor, 40 pg/ml tri-iodothyronine (Merck/Millipore Watford, Hertfordshire, UK), and 10% fetal calf serum (FCS; Greiner Bio-One, Alphen aan den Rijn, the Netherlands) [14, 33]. Posteriorly, the cell culture was maintained for 7 days at 37 °C and 5% v/v CO_2_, using the same medium composition to allow differentiation, expression of OAT1 and monolayer formation, referred to as maturation.

### Tubuloids culture

The tubuloids, derived from human cortical kidney tissue, were obtained from Hubrecht Organoid Technology (HUB), Utrecht, the Netherlands (OSR-2020-30b), and were cultured according to Schutgens et al. [10] Briefly, the tubuloids were maintained at 37 °C and 5% v/v CO_2_ in Basement Membrane Extract (BME) (R&D Systems, Abingdon, UK) and cultured in expansion medium (ADMEM/F12 supplemented with 1% penicillin/streptomycin, HEPES, GlutaMAX, N-acetylcysteine (1 mM; Sigma-Aldrich, Zwijndrecht, the Netherlands) and 1.5% B27 supplement (Gibco, Life Technologies, Paisley, UK), supplemented with 1% Rspo3-conditioned medium (U-Protein Express, Utrecht, The Netherlands), EGF (50 ng ml^–1^; Peprotech, London, UK), FGF-10 (100 ng ml^–1^, Peprotech, London, UK), Rho-kinase inhibitor Y-27632 (10 µM; Abmole, Brussels, Belgium) and A8301 (5 µM; Tocris Bioscience, Abingdon, UK)). For tubuloids differentiation, the medium was changed to ADMEM/F12 supplemented with 1% penicillin/streptomycin, HEPES and GlutaMAX, defined as differentiation medium and the tubuloids were maintained in culture for 7 days (Additional file 1).

### Conditioned medium (CM) production and EVs isolation

**Fig. 1:**
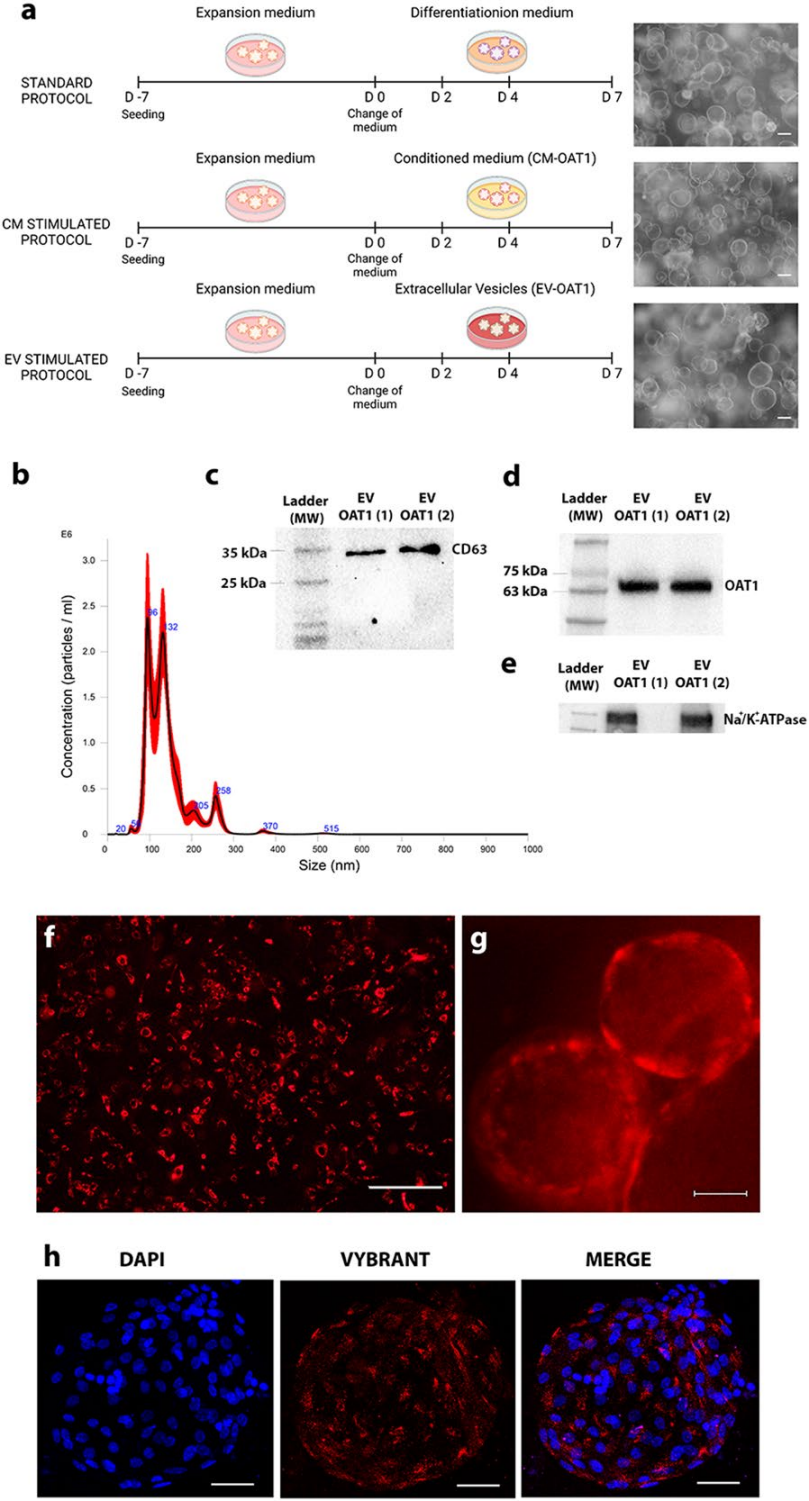
Characterization of EV-OAT1 and uptake by tubuloids. **a** Scheme of the tubuloid differentiation protocols used. The days (D) between D-7 and D0 comprehend the expansion phase of tubuloids. D0–D7 regards the expansion phase. D0, D2 and D4 indicate the days where new stimulation was given (with differentiation medium, CM-OAT1 or EV-OAT1). The images in the right show light microscopy representative images of tubuloid cultures in the different conditions. **b** Nanoparticle Tracking Analysis representative graph of EV-OAT1. The graph shows the size distribution of EVs (abscissa) and their concentration in particles/ml (ordinate). **c** Representative Western blot showing the presence of OAT1 within EV-OAT1 cargo. **d** Representative Western blot showing the presence of CD63 as exosome marker in EV-OAT1. **e** Representative Western blot showing the presence of Na^+^/K^+^-ATPase within EV-OAT1 cargo. **f** Fluorescence image of fully differentiated ciPTEC-OAT1 culture stained with Vybrant DiI (in red) (scale bar = 500 µm). **g** Fluorescence image showing the uptake of stained EV-OTA1 (in red) by tubuloids after 24 h incubation (scale bar = 50 µm). **h** Representative confocal image of EV-OAT1 distribution into tubuloids after 24 h incubation. The nuclei of the cells were stained with DAPI (in blue) and the EVs were stained with Vybrant DiI (in red) (scale bar = 50 µm)

After maturation of ciPTEC-OAT1, the cell culture was washed 3 times with Hanks’ Balanced Salt solution (HBSS; Gibco, Life Technologies, Paisley, UK) and maintained for 24 h at 37 °C and 5% v/v CO_2_ in tubuloid differentiation medium. The supernatant was collected, centrifuged at 3000×*g* at 4 °C and filtered under sterile conditions using a 0.22 µm filter (Millex-GV, PVDF; Merck/Millipore Watford, Hertfordshire, UK), and is referred to as CM-OAT1. To isolate the EVs from ciPTEC-OAT1, the CM-OAT1 was ultrafiltered using Amicon® Ultra-15 Centrifugal Filter Unit with 100 KDa cutoff (Merck/Millipore Watford, Hertfordshire, UK). The size and number of isolated EV-OAT1 and in the EVs present in CM before isolation were assessed by Nanoparticle tracking analysis using NanoSight NS500 (Malvern, Worcestershire, UK) with the following settings: camera CMOS, laser Blue 405, camera level 13 (NTA 3.0 Levels), slider shutter 800, FPS 25, number of Frames 1500 and temperature 24.5–24.6 °C (Fig. 1 and Additional file 4: Fig. S3). To determine the specificity of EV-OAT1 actions, the concentrated EVs were further isolated from the remaining medium using ExoQuick-TC (SBI System Bioscience, Palo Alto, CA, USA). The isolated EV-OAT1 were used for incubation with tubuloids with a final concentration equivalent to the CM (5 × 10^8^ particles/well). EVs analysis of RNA content, Western blotting (WB) and further characterization by proteomic analysis were also performed.

### Tubuloid differentiation with CM or EVs

Tubuloids initially cultured in 12-well plates in the presence of expansion medium were divided into 3 main experimental groups (Fig. 1a): (i) control condition (TUB CTR), tubuloids cultivated for 7 days in the presence of differentiation medium, changed every 48 h (standard protocol); (ii) tubuloids cultured with CM-OAT1 (TUB CM-OAT1) for 7 days with CM-OAT1 changed every 48 h (3 changes in total); (iii) tubuloids cultured with EV-OAT1 (TUB EV-OAT1) for 7 days in the presence of EVs derived from ciPTEC-OAT1 (5 × 10^8^ particles/well each stimulation), with every 48 h a new stimulation with EVs (3 stimuli in total: 15 × 10^8^ particles/well). To determine if the changes in tubuloids were given by EV direct transfer of cargo, changes in tubuloid gene expression or both mechanisms, additional conditions were added as follows: (iv) tubuloids stimulated on day 1 of differentiation with 3 doses of EV-OAT1 (15 × 10^8^ particles/well) on a single day and maintained in culture for 7 days (TUB EV-OAT1 3st*). Every 48 h the medium was changed with no additional EV-OAT1 treatment. Therefore, any EV-OAT1 that might have remained in the supernatant after 48 h was removed and longtime gene regulation would be associated strictly with changes in tubuloid gene expression, and not direct transfer. (v) tubuloids stimulated on day 5 of differentiation with 3 doses of EV-OAT1 (15 × 10^8^ particles/well) on a single day and maintained in culture until day 7 (TUB EV-OAT1 3st**). In this condition, no change of medium was performed after the administration of EV-OAT1. The purpose of this condition was to evaluate the OAT1 expression levels in tubuloids when the stimulation is given at the end of the differentiation protocol. In all experimental conditions, the tubuloids were cultured at 37 °C and 5% v/v CO_2_. After the 7 days of differentiation, tubuloids were harvested and centrifuged (500×*g* at 4 °C, 5 min). The pellet containing tubuloids was then used for further analysis (Additional file 5).

### Bioengineering tubuloid-derived kidney tubules

For kidney tubule engineering, microPES (polyethersulfone) hollow fiber membranes were used, sterilized with 70% (v/v) EtOH incubation for 30 min as described previously [16, 34, 35]. The fibers were coated with 10 mM L-3,4-dihydroxyphenylalanine (L-DOPA) (Sigma-Aldrich, MO, USA) dissolved in 10 mM Tris buffer (pH 8.5) at 37 °C for 5 h. A second layer of coating was given by fiber incubation with human collagen IV (C6745-1 ml, 25 μg·ml^−1^) for 1 h at 37 °C.

The tubuloids were dissociated in single cells as described above and seeded on double-coated fibers (length 2 cm) using 1 × 10^6^ cells/fiber and incubated at 37 °C and 5% v/v CO_2_ for 16 h. After, the unattached cells were removed, and the attached cells were maintained in culture with expansion medium until covering the entire surface of the fiber. Posteriorly, the cell on fibers were submitted to the differentiation protocol for the different experimental conditions (Fig. 1a).

### Statistical analysis

Statistical analyses were performed using Student t-test or one-way analysis of variance (ANOVA) test with Tukey’s post-test, using GraphPad Prism 5.0 (GraphPad Software, San Diego, CA USA). Statistical significance was set at P < 0.05 and data are expressed as mean ± SEM.

## Results

### Characterization of EVs and their uptake by the tubuloids

An initial characterization of EV-OAT1 was performed (Fig. 1a), revealing that mature ciPTEC-OAT1 secrete a heterogeneous population of EVs, ranging from 20 to 594 nm, with a mean value of 131 nm (Fig. 1b). Light microscopy images show similar morphology of tubuloids among the different protocols (Fig. 1a). A representative image of EV-OAT1 size distribution is presented in Fig. 1b. A parallel analysis was performed directly with the CM to evaluate the profile of EVs before isolation. EVs present in CM present a similar profile in size distribution when compared with the isolated EV-OAT1 (Additional file 4: Fig. S3). Western blotting of the EVs showed the presence of OAT1 amongst its cargo (Fig. 1c). Moreover, qRT-PCR analysis revealed the presence of mRNA of OAT1 (*SLC22A6*; 2^−ΔCt^: 3.27 ± 0.55, relative to the housekeeping gene HPRT1), indicating that OAT1 can be delivered into the tubuloids as mRNA and protein constructs. Proteomic analysis of EVs confirmed the presence of the exosome population by markers like CD9, CD63, CD81 and TSG101 [36] in the top 50 most expressed proteins in EVs in the EVpedia database (Table 1 and Additional file 6: Table S2), of which CD63 expression was also confirmed by Western blotting (Fig. 1d). Another important protein identified in the EVs is the Na^+^/K^+^-ATPase Transporting Subunit Alpha 1 (ATP1A), an enzyme encoded by the *ATP1A1* gene that is crucial for maintaining the electrochemical gradients of sodium and potassium ions across the plasma membrane. These gradients are essential for osmoregulation and for sodium-coupled transport of a variety of organic and inorganic molecules, including the transport of organic anions by OAT1 [37]. The abundance of Na^+^/K^+^-ATPase is also an indication of cell polarization [38]. Western blotting of the EV-OAT1 confirmed its presence (Fig. 1e).

**Table 1:** Top 50 proteins most expressed in the EVs identified in the EVpedia database

Index	Gene	Protein name	Identified	Index	Gene	Protein name	Identified
1	PDCD6IP	Programmed cell death 6-interacting protein	399	26	MSN	Zinc finger protein MSN2	266
2	GAPDH	Glyceraldehyde-3-phosphate dehydrogenase	377	27	ATP1A1	Na(+)/K(+) ATPase alpha-1 subunit	266
3	HSPA8	Heat shock cognate 71 kDa protein	363	28	PRDX1	Peroxiredoxin-1	263
4	ACTB	Actin, cytoplasmic 1	350	29	MYH9	Myosin-9	262
5	ANXA2	Annexin A2	337	30	EZR	Ezrin	262
6	CD9	CD9 antigen	328	31	CD81	CD81 antigen	262
7	PKM	Pyruvate kinase muscle isozyme	327	32	ANXA6	Annexin A6	260
8	HSP90AA1	Heat shock protein HSP 90-alpha	327	33	FLOT1	Flotillin-1	259
9	ENO1	Alpha-enolase	327	34	YWHAB	14-3-3 protein beta/alpha	258
10	ANXA5	Annexin A5	313	35	LDHB	L-lactate dehydrogenase B chain	258
11	HSP90AB1	Heat shock protein HSP 90-beta	306	36	SLC3A2	4F2 cell-surface antigen heavy chain	257
12	CD63	CD63 antigen	306	37	GNB1	Guanine nucleotide-binding protein G(I)/G(S)/G(T) subunit beta-1	257
13	YWHAZ	14-3-3 protein zeta/delta	301	38	PFN1	Profilin-1	256
14	YWHAE	14-3-3 protein epsilon	300	39	TSG101	Tumor susceptibility gene 101 protein	255
15	EEF1A1	Elongation factor 1-alpha 1	295	40	YWHAQ	14-3-3 protein theta	254
16	PGK1	Phosphoglycerate kinase 1	291	41	GNAI2	Guanine nucleotide-binding protein G(i) subunit alpha-2	252
17	CLTC	Clathrin heavy chain 1	283	42	CLIC1	Chloride intracellular channel protein 1	251
18	PPIA	Peptidyl-prolyl cis–trans isomerase A	278	43	ANXA1	Annexin A1	251
19	SDCBP	Syntenin-1	277	44	ITGB1	Integrin beta-1	250
20	ALDOA	Fructose-bisphosphate aldolase A	275	45	LDHA	L-lactate dehydrogenase A chain	249
21	EEF2	Protein-lysine N-methyltransferase EEF2KMT	274	46	FASN	Type I Fatty Acid Synthase	248
22	ALB	Albumin	274	47	CDC42	CDC42 small effector	248
23	TPI1	Triosephosphate isomerase	270	48	RAP1B	Ras-related protein Rap-1b	242
24	VCP	Transitional endoplasmic reticulum ATPase	269	49	CCT2	T-complex protein 1 subunit beta	242
25	CFL1	Cofilin-1	268	50	YWHAG	14-3-3 protein gamma	240

EVs isolated from Vybrant DiI-stained ciPTEC-OAT1 (Fig. 1f) were used to evaluate the uptake by tubuloids. After 24 h of incubation, the tubuloids internalized the vesicles (Fig. 1g), which were widely distributed in the tubuloids (Fig. 1h).

### CM-OAT1 and EV-OAT1 enhance the expression of transepithelial transporters and associated transcription factors

**Fig. 2:**
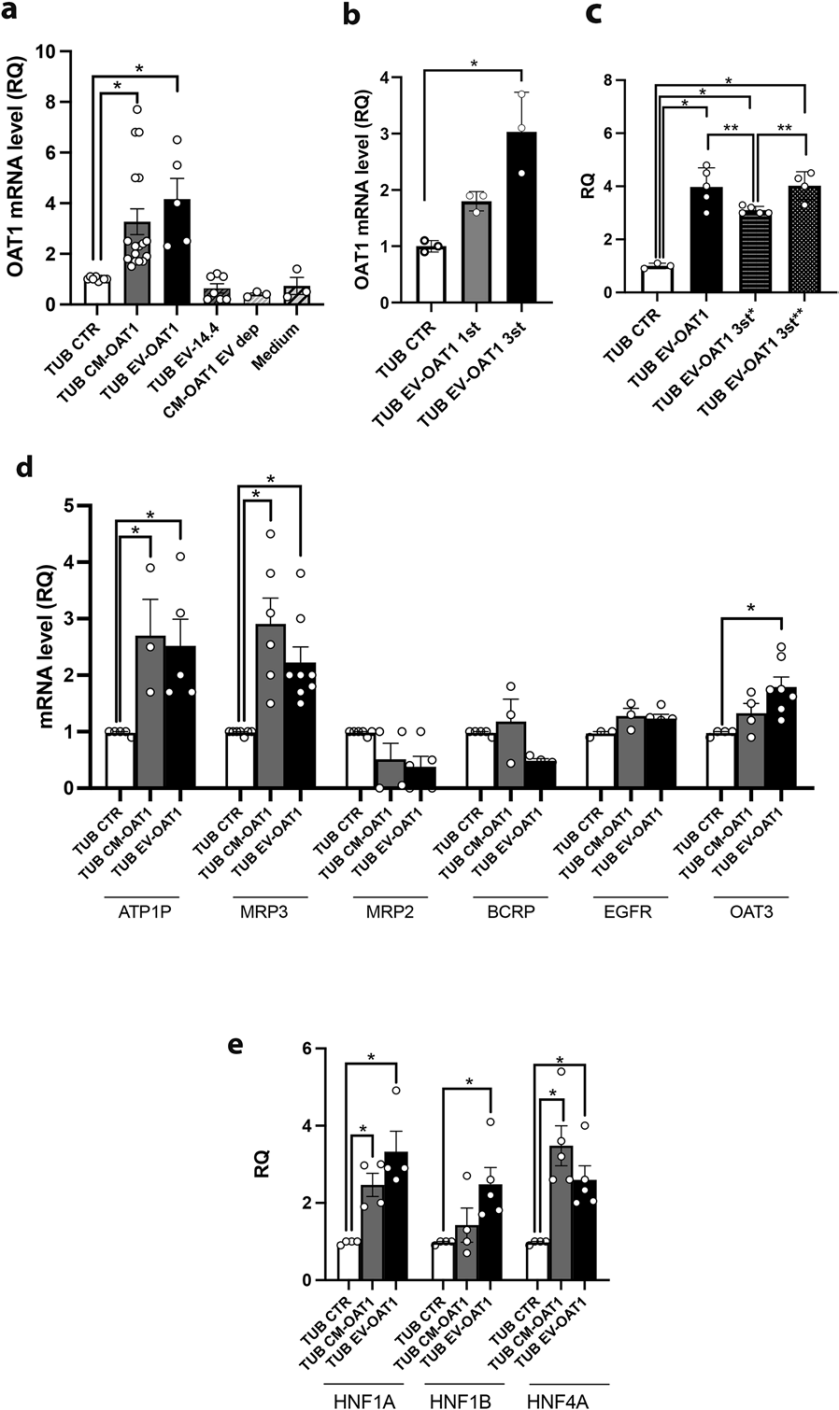
EV-OAT1 promote changes in epithelial transporters and transcription factors genes in tubuloids. **a** Conditioned medium and extracellular vesicles from ciPTEC-OAT1 (CM-OAT1 and EV-OAT1) upregulate OAT1 in tubuloids (TUB). The graph shows the changes in the mRNA levels in the tubuloids in different experimental conditions indicated in the abscissa (CTR represents standard differentiation protocol; EV-14.4 indicates EVs derived from ciPTEC 14.4; EV dep indicated CM-OAT1 depleted of EVs; Medium indicates the differentiation medium submitted to the same process of concentration for EVs and that was incubated with the tubuloids). **b** EV-OAT1 promote OAT1 upregulation in the tubuloids in a dose-dependent manner. The graph indicates the mRNA OAT1 levels stimulates with EV-OAT1 with a single dose (TUB EV-OAT1 1st) or three doses in a single stimulation (TUB EV-OAT1 3st). **c** The graph shows the changes in OAT1 mRNA levels in the tubuloids after different stimulation timepoints. The abscissa indicates the condition: TUB EV-OAT1 indicates normal stimulation protocol with administration of 3 doses in 3 different days within 7 days; 3st* indicates the stimulation of 3 doses in a singles stimulus in the beginning of differentiation protocol (day 1), while 3st** indicates the same single stimulus at the end of the protocol (day 5). **d** CM-OAT1 and EV-OAT1 promote the upregulation of other epithelial transporters in tubuloids. The graph shows the mRNA levels of epithelial transporters (ATP1, MRP3, MRP2, BCRP and OAT3) of tubuloids cultured in the different experimental conditions (abscissa). **e** Transcription factors associated with drug transport are upregulated by CM-OAT1 and EV-OAT1. The graph shows the mRNA levels of transcription factors in the tubuloids (HNF1A, HNF4A, HNF1B). In all graphs, the data is expressed in relative quantification (RQ) with respect to the control condition (TUB CTR) (n = 5). Data represent mean ± SEM, *p < 0.05 with respect to TUB CTR group and **p < 0.05 with respect to TUB EV-OAT1 3st* group

Evaluation of OAT1 expression in tubuloids revealed that CM-OAT1 induced OAT1 mRNA and protein levels, compared to control (Fig. 2a). The tubuloids did not present an increase in OAT1 mRNA when incubated with CM-OAT1 depleted of EVs confirming that the EVs and their cargo are responsible for the upregulation. To elucidate the mechanism involved in OAT1 upregulation, we exposed tubuloids to two different concentrations of EV-OAT1: (i) one stimulation (0.5 × 10^9^ particles/well; TUB EV-OAT1 1st) or (ii) three stimulations on a single day (3 × 0.5 × 10^9^ particles/well; TUB EV-OAT1 3st), in the first 24 h of tubuloids differentiation phase (Fig. 2b). The tubuloids stimulated with a single dose or three doses showed a 1.8- and three-fold increase at the OAT1 mRNA levels, respectively. This indicates a dose-dependent, but saturable delivery of EV-OAT1 cargo.

Furthermore, we evaluated if such modulatory effect in tubuloids could be maintained for a longer period after stimulation. After maintaining tubuloid cultures for 7 days upon a three doses stimulation at day 1 (TUB EV-OAT1 3st*), the levels of OAT1 were still higher compared to control condition (three-fold increase; Fig. 2c). However, the levels were lower than whenever the three doses were given during the 7 days of culturing (TUB EV-OAT1), even though the total dose of EV-OAT1 administrated was the same. But when the three doses are added at the end of differentiation protocol (TUB EV-OAT1 3st**), on day 5, the OAT1 levels were similar to the three doses along the 7 days and led to a four-fold increase. These results indicate that OAT1 upregulation mediated by EVs is not exclusively given by direct transfer. In addition, the stimulation performed closer to the end of the differentiation protocol may be more effective to induce the expression. It is worth to mention that the amount of OAT1 mRNA present inside the EVs is comparable to the increased level observed in the tubuloids after incubation with EV-OAT1 (Additional file 2: Fig. S1), arguing for direct transfer of OAT1 mRNA cargo.

Since ciPTEC-OAT1 overexpress the transporter, we evaluated the effects on tubuloids of EVs derived from the parent cells that do not express OAT1 (ciPTEC-14.4) [33]. Size distribution analysis of EVs derived from ciPTEC-14.4 (EV-14.4) showed a similar profile as EV-OAT1 (22–575 nm, with a mean value of 180 nm), but lack the capability of inducing OAT1 expression in tubuloids (Fig. 2a), indicating that the high expression of the transporter in ciPTEC-OAT1 is a vital element for the modulatory effect of EVs. Moreover, EV-OAT1 isolated with additional purification step using ExoQuick-TC also promoted the expression of OAT1 in the tubuloids (Additional file 3: Fig. S2), confirming that the effects are exclusively mediated by EVs.

Additionally, we analyzed the mRNA levels of other transporters linked to OAT1 function and associated with the excretion of metabolic waste and drug handling, including *ATP1A*, *ABCC2* (MRP2) and *ABCC3* (MRP3), *ABCG2* (BCRP) and *SLC22A8* (OAT3; Fig. 2d). Of these transporters, *ATP1A* and *ABCC3* were equally increased by CM-OAT1 and EV-OAT1 when compared to control, whereas the expression levels for *ABCC2* and *ABCG2* were not altered. Interestingly, exposure of tubuloids to EV-OAT1 resulted in an increase in OAT3 mRNA levels, which was not observed in CM-OAT1 exposures. However, the increase in OAT3 was not as pronounced as observed for OAT1. Moreover, BCRP and MRP2 tended to be downregulated in the EV-OAT1condition, although not significantly different.

We further analyzed the expression of some transcriptional factors known to regulate tubule epithelial cell maturation and the expression of epithelial transporters [39] (Fig. 2e). The mRNA levels of hepatocyte nuclear factor 1 alpha, 4 alpha and 1 beta (*HNF1A*, *HNF4A* and *HNF1B*) revealed that CM-OAT1 and EV-OAT1 could regulate these genes in the tubuloids. CM-OAT1 promoted the expression of *HNF1A* and *HNF4A*, but not *HNF1B*, different from EV-OAT1that led to the upregulation of all three transcription factors.

### CM-OAT1 and EV-OAT1 improve tubuloid OAT1 protein expression, localization and transport capacity

**Fig. 3:**
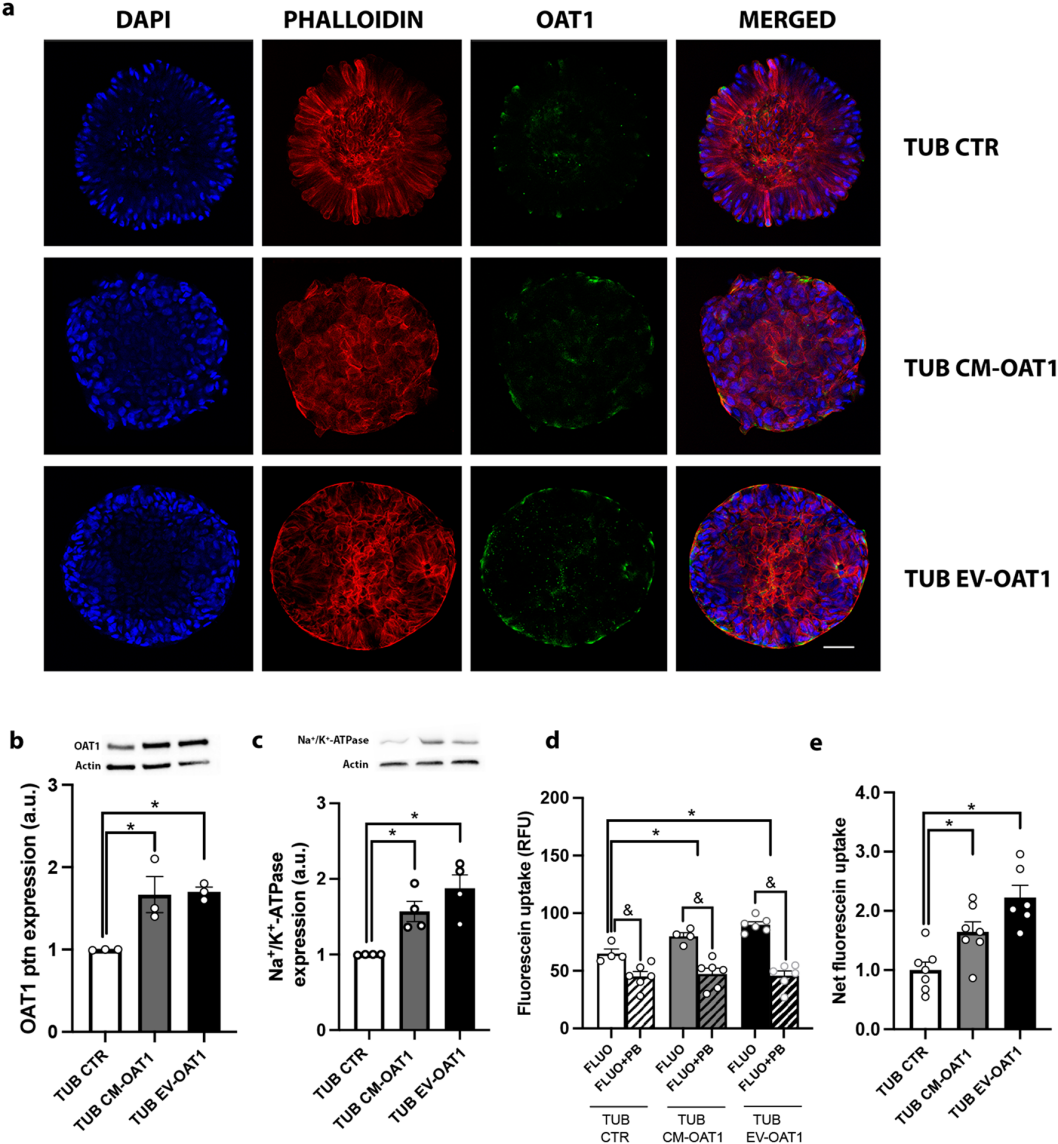
EV-OAT1 and CM-OAT1 support functional maturation of tubuloids. **a** Representative confocal images of OAT1 localization in the tubuloids under different experimental conditions. The nuclei of the tubuloid cell were stained with DAPI (in blue). The tubuloid spatial organization is indicated by actin disposition, stained with phalloidin (in red). OAT1 localization is observed by the green staining. The last column represents the merge of the three images of each respective experimental condition (scale bar = 50 µm). **b** OAT1 protein expression in the tubuloids. Upper panel shows representative images of Western blot for OAT1 and Actin. The graph shows the quantification of OAT1 expression after Western blotting (n = 3). **c** Na^+^/K^+^-ATPase protein expression in the tubuloids. Upper panel shows representative images of Western blot for Na^+^/K^+^-ATPase and Actin. The graph shows the quantification of Na^+^/K^+^-ATPase expression after Western blotting (n = 3). **d** Fluorescein uptake capacity by the tubuloids. The graph shows the intracellular fluorescence intensity of tubuloids after 10 min incubation with fluorescein (FLUO), in the presence or absence of probenecid (PB) (OATs inhibitor) (n = 4). The fluorescence intensity is expressed as arbitrary units (a.u.). **e** Net fluorescent uptake specific to OAT in tubuloids. The graph shows the increase of fluorescein uptake of tubuloids cultured with CM-OAT1 or EV-OAT1 in respect to tubuloids under standard differentiation condition. The specificity of transport was given by the difference in the fluorescence intensity between the presence and absence of probenecid. The data is presented as ration in respect to TUB CTR condition (n = 4). In all graphs, data represent mean ± SEM, *p < 0.05 compared to TUB CTR group, ^&^p < 0.05 compared to FLUO + PB for each experimental condition

The localization of OAT1 in the basolateral membrane is crucial for the vectorial transport of substrates such as metabolic wastes, but it is also an indication of epithelial cell polarization. Immunostaining of tubuloids after exposure to CM-OAT1 and EV-OAT1 demonstrated an increased polarized expression of OAT1 in the basolateral membrane of the cells, facing the outer part of the tubuloids (Fig. 3a). The tubuloids cultured under standard differentiation condition presented a more disperse localization of OAT1, although some basolateral foci of OAT1 can be observed as well. Quantification by Western blotting revealed that CM-OAT1 and EV-OAT1 induced an increase in OAT1 protein (Fig. 3b), but also in Na^+^/K^+^-ATPase (Fig. 3c), confirming a polarized maturation of the tubuloids. No significant changes were observed in actin levels among the experimental conditions.

Increased expression of OAT1 at the basolateral membrane should ultimately lead to an increase in the functional capacity of the tubuloids to transport organic anions. To this end, we evaluated the transport efficiency through fluorescein uptake by the tubuloid cells (Fig. 3d, e) and demonstrated an increase in the intracellular fluorescence intensity when compared to control (Fig. 3d). This was sensitive to probenecid, a known OAT1 inhibitor, confirming active transporter-mediated uptake. Uptake was more pronounced when tubuloids were matured in the presence of EV-OAT1, supporting EVs’ potential to functionally mature tubuloids (Fig. 3e). It is worth mentioning that OAT3 may also facilitate probenecid-sensitive fluorescein transport, but with a lower affinity [18]. The increased OAT1 expression in tubuloids cultured with CM-OAT1 together with the absence of changes in the OAT3 mRNA levels (Fig. 2b), however, argue for the improvement in transport capacity exclusively by OAT1.

### EVs drive cell maturation processes in tubuloids

**Fig. 4:**
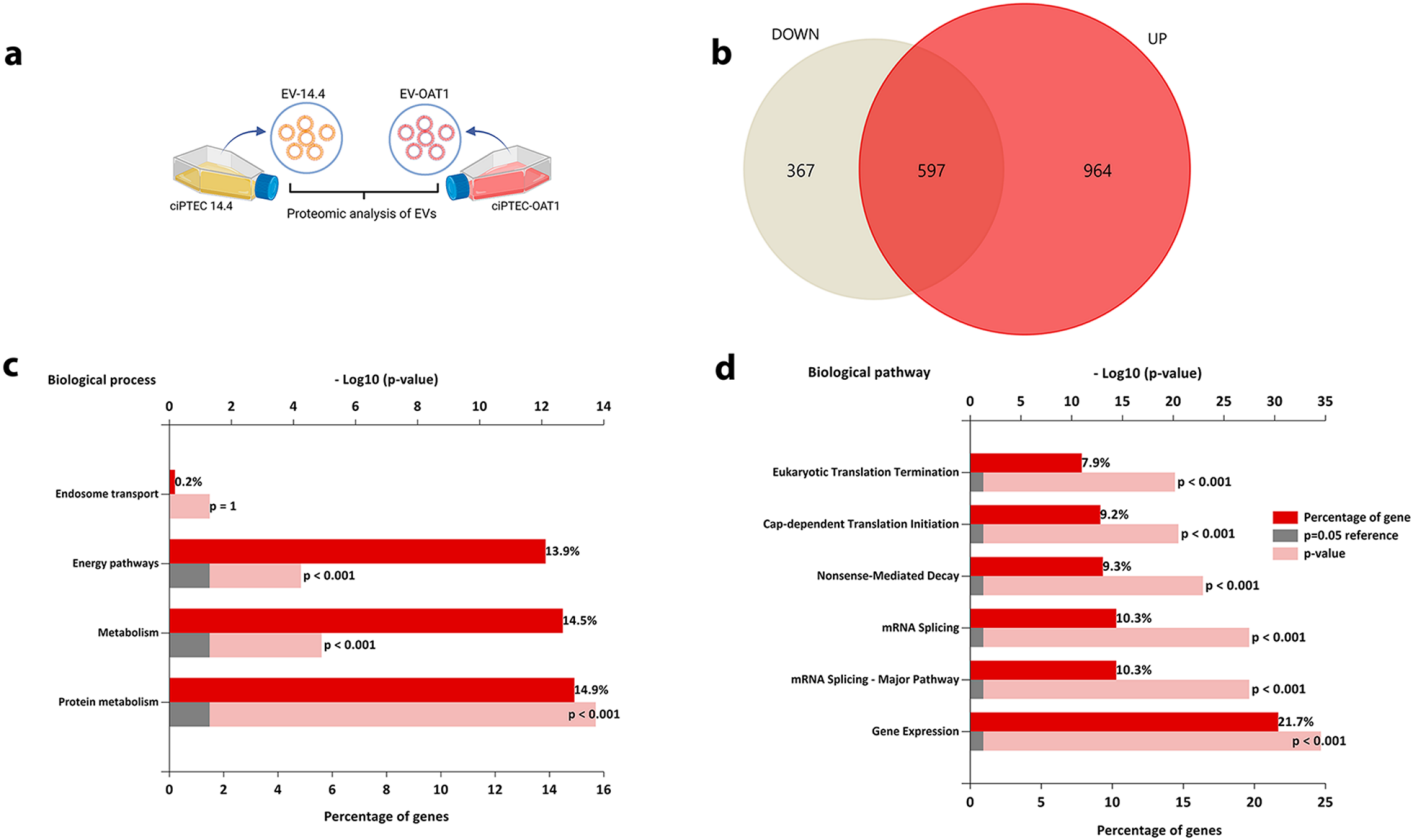
Proteomic analysis of EV-OAT1. **a** Representative scheme of the comparison of proteins presents in EV-OAT1 and EV-14.4. **b** The Venn diagram shows the proteins that are exclusively present or upregulated in EV-OAT1 (UP), commonly expressed and absent or downregulated in EV-OAT1 (DOWN) compared to EV-14.4. **c** The graph indicates the biological processes associated with the exclusively present or upregulated proteins in EV-OAT1. **d** The graph indicates the biological pathways associated with the exclusively present or upregulated proteins in EV-OAT1. The abscissa indicates − Log10(p-value)

Proteomic profile of EV-OAT1 and EV-14.4 (Fig. 4a, Additional file 6: Table S2) identified 964 proteins upregulated or exclusively expressed in EV-OAT1, 367 proteins upregulated or exclusively present in EV-14.4 (also defined as downregulated in EV-OAT1) and 597 proteins commonly expressed in both EVs (Fig. 4b). Functional enrichment analysis of the genes associated with the upregulated proteins in EV-OAT1 indicated that the identified proteins are mainly associated with protein metabolism, cellular metabolism and energy pathways (Fig. 4c). Moreover, the biological pathways associated with the upregulated proteins showed a regulatory capacity of EVs to modulate gene expression (e.g., mRNA splicing, translation initiation and termination (Fig. 4d)).

**Fig. 5:**
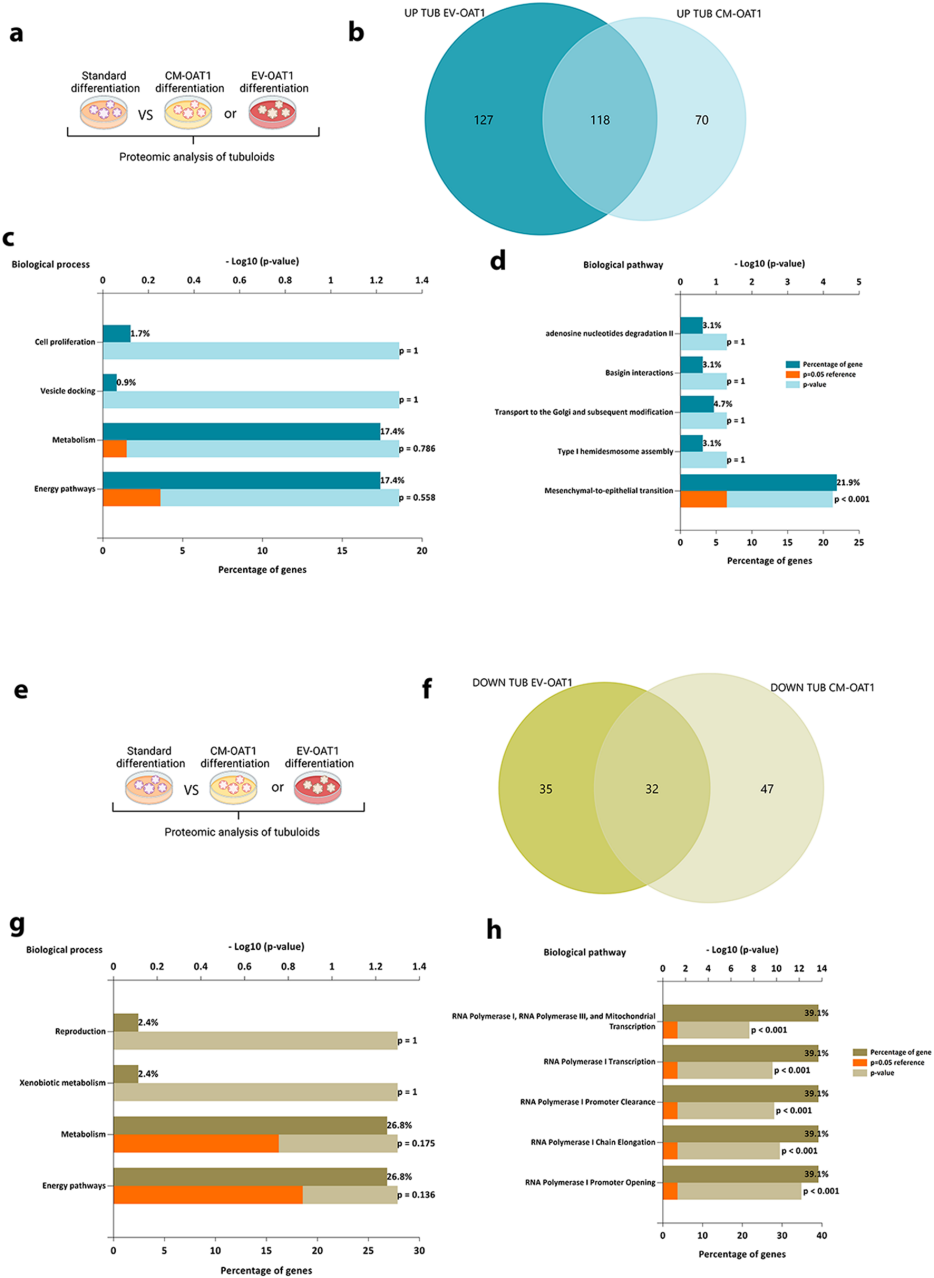
Pathways involved in the tubuloid changes promoted by CM-OAT1 and EV-OAT1. **a** Representative scheme of the comparison of proteins exclusively upregulated in tubuloids cultured with CM-OAT1 or EV-OAT1. **b** The Venn diagram shows the upregulated proteins in the tubuloids incubated with CM-OAT1 or EV-OAT1 as compared to tubuloids cultured under standard differentiation protocol. **c** The graph indicates the biological processes associated with the commonly upregulated proteins in tubuloids cultured with CM-OAT1 or EV-OAT1. **d** The graph indicates the biological pathways associated with the commonly upregulated proteins in tubuloids cultured with CM-OAT1 or EV-OAT1. The abscissa indicates − Log10(p-value). **e** Representative scheme of the comparison of proteins exclusively downregulated in tubuloids cultured with CM-OAT1 or EV-OAT1. **f** The Venn diagram shows the downregulated proteins in the tubuloids incubated with CM-OAT1 or EV-OAT1 compared to tubuloids cultured under standard differentiation protocol. **g** The graph indicates the biological processes associated with the commonly downregulated proteins in tubuloids cultured with CM-OAT1 or EV-OAT1. **h** The graph indicates the biological pathways associated with the commonly downregulated proteins in tubuloids cultured with CM-OAT1 or EV-OAT1. The abscissa indicates − Log10(p-value)

Next, the proteome of tubuloids (Fig. 5, Additional file 7: Table S3, Additional file 8: Table S4, Additional file 9: Table S5) showed that 188 and 245 proteins were upregulated in the tubuloids cultured with CM-OAT1 and with EV-OAT1, respectively, when compared to control (Fig. 5a, b). Among the identified proteins, 118 were shown to be commonly upregulated in tubuloids cultured with CM-OAT1 and EV-OAT1, including neutrophil gelatinase-associated lipocalin (Lcn2/NGAL) and Plexin B2 (Plxnb2) known regulators of kidney maturation [40, 41]. Functional enrichment analysis of the genes associated with these proteins revealed that they are mainly associated with energy pathway and cellular metabolism (Fig. 5c), similar to those observed in the EVs proteomic analysis. Notably, the mesenchymal-to-epithelial transition (MET) is one of the key pathways triggered by EVs (Fig. 5d).

Furthermore, 79 proteins were downregulated in tubuloids cultured with CM-OAT1 and 67 proteins in the presence of EV-OAT1 (Fig. 5e, f). The common 32 downregulated proteins were mainly associated with cellular metabolism and energy supply, although not statistically significant (Fig. 5g). The biological pathways associated with the reduced proteins indicate a strong relation with the regulation of RNA transcription (e.g., RNA polymerase I promoter opening, chain elongation and transcription) (Fig. 5h). Among the downregulated proteins, the histone H3 subunits were reduced mostly by EVs, indicating a role in regulation of chromatin organization and DNA replication (Additional file 8: Table S4) [42].

### EV-treated tubuloids form polarized kidney tubules on hollow fiber scaffolds

**Fig. 6:**
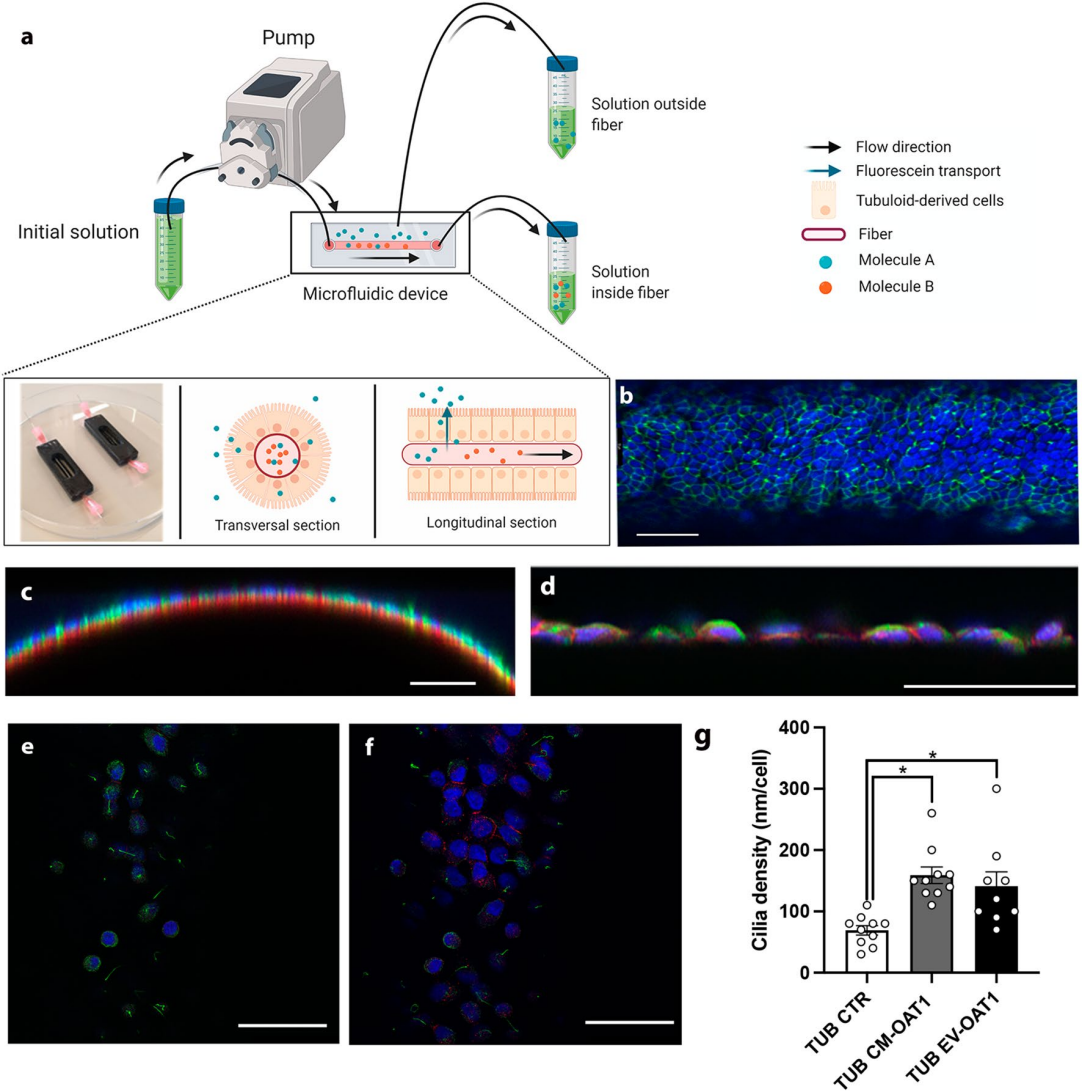
Tubuloid-derived cell form tight monolayer on microfluidic hollow fiber-based platform and CM-OAT1 and EV-OAT1 improve cilia density. **a** Scheme of the microfluidic hollow fiber-based platform and how tubuloid-derived cells are organized, and selectively transport molecules from one compartment to another. **b** Representative confocal image of tubuloids-derived cells cultured on hollow fibers in the presence of EV-OAT1. The ZO-1 immunostaining (in green) shows the presence of a tight epithelial monolayer on the fiber. **c** Representative y–z confocal image of the curved surface of the fiber and the polarized tubuloid-derived cells cultured with EV-OAT1. The acetyl-α-tubulin immunostaining in the apical region (in green) indicates the cilia structure formation and Na^+^/K^+^-ATPase is localized to the basolateral region (in red). **d** Higher magnification of tubuloids cells on the fibers with α-tubulin (in green) and Na^+^/K^+^-ATPase (in red) staining. **e** The cilia structures (in green) are mainly observed when the apical region is in focus, while in **f** the presence of Na^+^/K^+^-ATPase (in red) is mainly observed in the basolateral region. In all images, scale bar = 50 µm. **g** Quantification of total cilia perimeter present in the tubuloids-derived cells under the different experimental conditions. The graph shows the total cilia perimeter with respect to the total number of cells. Data represent mean ± SEM, *p < 0.05 compared to TUB CTR group

One of the potential applications of tubuloids in regenerative medicine is the creation of functional units in a bioartificial kidney. Here, we evaluate if EVs could support the use of tubuloid-derived cells to bioengineered kidney tubules (Fig. 6a). Immunofluorescent staining of the tight-junction protein ZO-1 showed that the tubuloid-derived cells established a tight 3-dimensional monolayer, with no differences among the three experimental conditions (Fig. 6b). Furthermore, localization of Na^+^/K^+^-ATPase in the basolateral membrane and cilia structure formation at the apical region confirmed adequate polarization of the tubuloid-derived monolayers (Fig. 6c–f). Again, culturing the kidney tubules in the presence of CM-OAT1 or EV-OAT1 enhanced the cell polarity process as indicated by an increased cilia density determined by the ratio of the cilia total perimeter and the number of cells (Fig. 6g).

## Discussion

EVs have been described as an essential element in intranephron communication and kidney regeneration [43]. In this study, we evaluated a novel role for EVs as molecular messengers for functional maturation of kidney tubular organoids in vitro. We showed that EVs derived from mature kidney cells overexpressing OAT1 (EV-OAT1) improved the tubuloids phenotype by triggering various biological pathways associated with epithelial maturation. As a result, tubuloids showed enhanced polarization, increased formation of cilia structures, and improved epithelial transport capacity with increased mRNA levels of several proximal tubular transporters, including OAT1. The evolutionarily well conserved solute carrier coordinates the active secretion of a broad range of endogenous and exogenous substances. Unfortunately, the transporter is rapidly lost in primary kidney cell cultures and not functionally present in tubuloids. The use of EVs provide a tool to induce the transporter expression, which amplifies the tubuloids applications. Concomitantly, *HNF1A*, *HNF4A* and *HNF1B* were upregulated, three master regulatory transcription factors towards kidney lineage differentiation [44], kidney tubular epithelial maturation [45] and xenobiotic transporters, including OAT1 and OAT3 [46–48]. It should be noted, however, that OAT1 upregulation in tubuloids was much more pronounced upon CM and EV exposure than OAT3, which is likely due to the high expression levels of the transporter in ciPTEC-OAT1, whereas OAT3 is absent in this cell line. Western blotting and qRT-PCR analysis showed that EV-OAT1 cargo contains OAT1 as both mRNA and as protein. Therefore, a direct transfer of OAT1 cargo can drives the upregulation of OAT1, although indirect transcriptional regulation should not be excluded as OAT1 upregulation is still observed after several days after EV stimulation and OAT3 was also upregulated in the tubuloids exposed to EV-OAT1 but not CM-OAT1. Future experiments using e.g., RNA polymerase inhibitors, such as alfa-amanitin, should shed light on the mechanism of OAT1 induction by distinguishing between direct transfer or induction.

The observed upregulation of Na^+^/K^+^-ATPase is of great importance for functional OAT1 as organic anion transport is tertiary coupled to the sodium gradient generated by this pump [49]. Again, it remains to be elucidated whether the joint upregulation of OAT1 and Na^+^/K^+^-ATPase is mediated indirectly by induced regulatory pathways or through a direct transfer of mRNA and protein, or both. Besides proximal tubule cells, tubuloids also contain cells from the distal tubule, loop of Henle, and collecting duct epithelium, which take up EVs [10]. Interestingly, EV exposure increased the expression of the basolateral transporter MRP3, an efflux pump expressed in kidney distal tubule cells [48], indicating that EV-OAT1 also promoted changes in cell types other than proximal tubule cells. This argues for EVs mediating cell responses not only via direct cargo transfer as MRP3 is not expressed in the originator cells. Additionally, the expression of other transporters like MRP2 and BCRP were not modified or slightly reduced, indicating a selectivity modulation in gene expression or that the EVs may not present all information to modify all proteins belonging to the epithelial transport machinery.

Proteomic analyses showed that the main biological processes modulated upon CM and EV exposures were energy pathways and cellular metabolism, similar to processes found in EV’s cargo analysis, which supports the relation between EV cargo composition and the response triggered in the target cells. Furthermore, pathway analysis indicated that mesenchymal-to-epithelial transition is one of the main pathways activated, which supports the phenotypic improvements observed (e.g., cellular polarization and increased cilia density). Among the 964 upregulated proteins in tubuloids incubated with EVs, we identified neutrophil gelatinase-associated lipocalin (Lcn2/NGAL) that has been described to facilitate iron delivery into cells. Lcn2 regulates iron-sensitive genes that participate in mesenchymal-to-epithelial transition during the development of the proximal parts of the mammalian nephron [40, 50]. Additionally, Plexin B2 (*Plxnb2*), a semaphorin receptor expressed in the pretubular aggregates and the ureteric epithelium in the developing kidney, was found upregulated [51]. Plxnb2-deficient mice were shown to present intrinsic defects of the ureteric epithelium, leading to reduced branching and proliferation. Moreover, this receptor is directly associated with the formation and organization of the collecting duct, a nephron segment that is also present in the tubuloids [10, 41]. One of the most downregulated proteins upon EV exposure was histone H3. Histone H3 synthesis is coupled to DNA replication, providing material for the bulk of nucleosome assembly for the duplicated genome [42]. Thus, downregulation of histone H3 indicates a reduction in tubuloid cell proliferation. This is supported by the reduced expression of the proliferating cell nuclear antigen (PCNA), which is rapidly downregulated when kidney epithelial cells enter terminal differentiation and acquire functional characteristics during kidney development [52].

EVs have been investigated for kidney disease treatment as regulators of several processes, including counteracting inflammatory responses, as anti-oxidative stress response, and prevention of cell death [23, 53–56]. In this study, we demonstrated a new application potential for EVs in regenerative nephrology by supporting tubuloid maturation on a hollow fiber-based microfluidics device to be used as functional unit of a bioartificial kidney. Previously, such device has been shown to successfully recreate aspects of kidney tubular function by establishing a polarized monolayer capable of promoting active waste excretion using ciPTEC-OAT [34, 57]. The immortal nature of these cells, harboring two oncogenes, however, hamper their future clinical application in kidney replacement therapies. This limitation can be overcome using tubuloids if their maturation can be supported. The present results show that tubuloids can be matured by EVs and used to form a tight epithelial monolayer on hollowfiber membranes with enhanced polarization given by increased cilia structure density. Cilia are sensory organelles located at the apical membrane that sense fluid flow and initiate calcium-based signaling to regulate tubular function and maintain an epithelial phenotype [58, 59]. Moreover, cilia structures have been shown to play a critical role in the Hedgehog and Wnt signalling pathways that are also associated with epithelial development [60, 61]. These findings indicate that EVs can be a key tool for the maturation of tubuloids and kidney tubule engineering. It is worth mentioning that, despite similar effects, CM and EVs can present distinct outcomes. The advantage of using of EVs over CM is that it presents an improved strategy to deliver molecules with more control of the content and stability of the molecules. By bioengineering EVs with a finetuned cargo, [23, 62] tubuloids might be phenotypically modulated even further in the future to approach a near-physiological phenotype found in native tissue. In this manner, the EVs may represent a key element in the development of bioartificial kidneys with cells derived from tubuloids or organoids, guiding the differentiation and maturation process of multiple kidney cell types. The clinical translational, however, remais a long way. Still, advances in understanding the extracellular matrix composition, vascularization, microfluidics and other elements associated to the kidney microenvironment may advance the application of tubuloids and kidney organoids in renal replacement therapies.

### Conclusion

In conclusion, EVs from kidney tubular epithelial cells are capable of changing tubuloid’s phenotype, acting in different elements of tubule functional maturation and can be considered an additional factor to support the kidney microenvironment in vitro. Such innovative approach advances preclinical models’ development and supports applications in modelling tubular function in health and disease and in renal replacement therapies. Moreover, the use of EVs can be extended to other tissue models to be applied as a strategy to support the maturation of organoids derived from adult stem cells and iPSCs. Finally, we believe that a better understanding of the mechanisms involved in the regulation mediated by EVs (direct transfer or transcriptional regulation) and the identification of regulatory molecules with which to bioengineer EVs, offer translational tools to control organoid fate in a patient-specific manner.

## Supplementary Information

**Additional file 1: Methods S1.**

**Additional file 2: Figure S1.** OAT1 mRNA levels within EV-OAT1 is compatible with the increased levels in tubuloids. The graph shows the total OAT1 mRNA levels present in EV-OAT1 and tubuloids that were previously isolated separately and then pooled to perform the qRT-PCR (TUB + EV-OAT1 MIX). The expression levels were compared to unstimulated tubuloids (TUB CTR). The data is expressed in relative quantification (RQ) in respect to the control condition (TUB CTR) (n = 3). Data represent mean ± SEM, *p < 0.05 with respect to TUB CTR group.

**Additional file 3: Figure S2.** EV-OAT1 additionally purified from the CM maintained the upregulation of OAT1 in tubuloids. The graph shows the changes in the mRNA levels in the tubuloids culture under standard differentiation protocol (TUB CTR) and in the presence EV-OAT1 that were further isolated from the remaining medium using ExoQuick-TC. The data is expressed in relative quantification (RQ) in respect to the control condition (TUB CTR) (n = 3). Data represent mean ± SEM, *p < 0.05 with respect to TUB CTR group.

**Additional file 4: Figure S3.** Nanoparticle Tracking Analysis representative graph of the EVs present in CM-OAT1, without isolation. The graph shows the size distribution of EVs (abscissa) and their concentration in particles/ml (ordinate).

**Additional file 5: Table S1.** List of primers.

**Additional file 6: Table S2.** List of all proteins identified in EV-OAT1 and its expression levels in respect to EV-14.4.

**Additional file 7: Table S3.** List of all proteins identified in TUB CM-OAT1 and its expression levels in respect to TUB CTR.

**Additional file 8: Table S4.** List of all proteins identified in TUB EV-OAT1 and its expression levels in respect to TUB CTR.

**Additional file 9: Table S5.** List of all proteins identified in TUB EV-OAT1 and its expression levels in respect to TUB CM-OAT1.

## Data Availability

All data generated and analyzed during this research are included in this published article. The mass spectrometry proteomics data have been deposited to the ProteomeXchange Consortium via the PRIDE partner repository with the dataset identifier PXD027142.
