# Supplementary material for: Differentiated kidney tubular cell-derived extracellular vesicles enhance maturation of tubuloids
Source: J Nanobiotechnology. 2022 Jul 15;20:326. doi: 10.1186/s12951-022-01506-6 (PMC9284832; doi:10.1186/s12951-022-01506-6)
Supplement: Supplementary file 1 — Additional file 1: Methods S1. [file 12951_2022_1506_MOESM1_ESM.docx]

**Additional file**

**Methods S1**

**EVs uptake by tubuloids**

To evaluate the EVs incorporation by tubuloids, matured ciPTEC-OAT1 were labelled with Vybrant® DiI (Thermo Fisher Scientific, Vilnius, Lithuania) and EVs, also labelled, were isolated from the supernatant of these cells.^1,2^ The tubuloids were incubated for 24 h with the labelled EVs. After, the tubuloids still inside the BME were washed 3 times with cold HBSS followed by a 20 min incubation with Dispase solution (StemCell Technologies, Cambridge, UK) at 37°C and 5% v/v CO_2_ to remove the BME around the tubuloids. The tubuloids were then fixed with 4% paraformaldehyde. Nuclei were stained with ProLong^TM^ Gold antifade reagent containing DAPI (Life Technologies, Eugene, OR, USA). The kidney EV incorporation was analyzed by inverted fluorescence microscopy (Nikon Eclipse Ts2, Melville, NY) for fresh cultures. For fixed cultures, images were acquired using confocal microscopy (Leica TCS SP8 X, Leica Biosystems, Amsterdam, The Netherlands) using the software Leica Application.

**Protein purification and mass spectrometry by RP-NanoLC–MS/MS**

Protein purification and digestion for proteomic analysis were initially given by lysing the samples in a detergent-based buffer – 1% sodium deoxycholate (SDC), 10 mM Tris (2-carboxyethyl) phosphine (TCEP), 10 mM Tris, 40 mM chloroacetamide) – with Complete mini EDTA-free protease inhibitor cocktail (Roche, Woerden, the Netherlands). The samples were boiled for 5 min at 95°C, 50 mM ammonium bicarbonate was added, and digestion was allowed to proceed overnight at 37°C using trypsin (Promega, Madison, WI, USA) and LysC (Wako, Richmond, VA, USA) at 1:50 and 1:75 enzyme:substrate ratios, respectively. The digestion was quenched with 10% formic acid and the resulting peptides were purified using the Oasis PRiME HLB system (Waters, Wilmslow, UK). Peptides were dried entirely and then resolubilised in a 2% formic acid (FA) MS loading buffer.

The proteomic analysis was performed by Reversed-Phase Liquid Chromatography Mass Spectrometry (RP-NanoLC–MS/MS). Data were acquired using an Ultimate3000 system (Thermo Fisher Scientific, Vantaa, Finland) coupled to an Orbitrap Q Exactive HF-X mass spectrometer (Thermo Fisher Scientific, Vantaa, Finland). Peptides were first trapped (Acclaim PepMap100 C18, 5 μm, 100A; Thermo Fisher Scientific, MA, USA) before being separated on an analytical column (Agilent Poroshell, CA, USA; EC-C18, 2.7 μm, 50 cm × 75 μm; Agilent). Trapping was performed for 2 min in solvent A (0.1 M FA in water), and the gradient was as follows: 9–13% solvent B (0.1 M FA in 80% ACN) in 3 min, 13-44% in 95 min, 44–95% in 3 min, and finally 100% for 4 min. The mass spectrometer was operated in data-dependent mode. Full-scan MS spectra from m/z 375–1,600 were acquired at a resolution of 60,000 at m/z 400 after accumulation to a target value of 3 × 10^6^. Up to 15 most intense precursor ions were selected for fragmentation. HCD fragmentation was performed at a normalized collision energy of 27 after accumulation to a target value of 1 × 10^5^. MS/MS was acquired at a resolution of 30,000.

For data analysis, raw mass spectrometry data files were searched using MaxQuant v. 1.6.17.0, against the human Uniprot protein database using Andromeda as a search engine.^3^ Cysteine carbamidomethylation was set as a fixed modification and methionine oxidation, protein N-term acetylation. Trypsin was specified as enzyme and up to two miss cleavages were allowed. Filtering was done at 1% false discovery rate (FDR) at the protein and peptide level. Label-free quantification (LFQ) was performed and quantified data were processed and analyzed using R and Perseus.^4^ The mass spectrometry proteomics data have been deposited to the ProteomeXchange Consortium via the PRIDE^5^ partner repository with the dataset identifier PXD027142". Enrichment analysis related to the identified proteins were performed with FunRich software.^6^

**Immunofluorescent staining**

Tubuloid-derived cell cultures in 6-well plates or fibers were fixated with paraformaldehyde 4% for 15 min. In the case of 3D conformation, the tubuloids were initially incubated with Dispase to remove the BME followed by fixation with paraformaldehyde 4%. The cells were permeabilized in 0.3% (v/v) triton X-100 in HBSS for 10 min and incubated with blocking solution containing 2% (w/v) bovine serum albumin fraction V (Roche, Woerden, The Netherlands) and 0.1% (v/v) tween-20 in HBSS for 30 min. The cells were incubated with primary antibodies diluted in block solution against OAT1 (ab135925, Abcam), the tight junction protein zonula occludens 1 (ZO-1; ab216880, Abcam), Na^+^/K^+^-ATPase (kindly provided by Dr JB Koenderink, Radboudumc, Nijmegen, the Netherlands^7^), acetylated α-tubulin (T6793, Sigma-Aldrich, MO, USA). The secondary antibodies used were the anti-rabbit-Alexa488 and the anti-mouse-Alexa-594 conjugates (Life Technologies Europe BV, Bleiswijk, The Netherlands). Phalloidin-iFluor 488 Reagent (ab176753) was used to evaluate F-actin expression. Nuclei were stained with ProLongTM Gold antifade reagent containing DAPI (Life Technologies Europe BV, Bleiswijk, The Netherlands). Images were obtained using confocal microscopy (Leica TCS SP8 X, Leica Biosystems, Amsterdam, The Netherlands) and the software Leica Application. To measure the cilia density, Multiple Z stacks images from immunofluorescent staining for acetylated α-tubulin were analyzed with ImageJ software to measure the total perimeter.^8^

**RNA isolation and quantitative real-time polymerase chain reaction (qRT-PCR)**

mirVana RNA isolation kit (Thermo Fisher Scientific, MA, USA) was used for RNA extraction from tubuloid and EV-OAT1. RNA quantification was measured spectrophotometrically using NanoDrop™ OneC Spectrophotometer (Thermo Fisher Scientific, MA, USA) mRNA expression was assessed using a High-Capacity cDNA Reverse Transcription Kit (Applied Biosystems) and iQ SYBR Green Supermix (Bio-Rad, Hercules, CA, USA). Negative cDNA controls (no cDNA) were cycled in parallel with each run. qRT-PCR was done with a CFX96 Real-Time PCR Detection System (Bio-Rad). All the sequence-specific oligonucleotide primers were obtained from Biolegio (Nijmegen, The Netherlands). See Table S1 for primers sequences.

**Western blotting**

Protein extraction from tubuloid and EVs was performed using Ripa Buffer supplemented with Halt™ Protease and Phosphatase Inhibitor Cocktail (Thermo Fisher Scientific, MA, USA). The protein concentration was measured using Pierce™ BCA Protein Assay Kit and GloMax® Explorer Multimode Microplate Reader (Promega, Madison, WI, USA). Expression of OAT1, Na^+^/K^+^-ATPase and CD63 were measured by Western blotting. The OAT1 antibody (ab131087) was purchased from Abcam (Cambridge, UK), Na^+^/K^+^-ATPase (given by Dr JB Koenderink)^9^ and CD63 (sc-5275) was purchase from Santa Cruz Biotechnology (Dallas, TX, USA). The β-actin antibody was used as a loading control (ab8226, Abcam). The secondary antibodies used were goat anti-rabbit and rat anti-rabbit IgG-HRP (Dako, Santa Clara, CA, USA). Proteins were detected by chemiluminescence using the Clarity™ Western ECL Substrate coupled to a ChemiDoc XRS+(Bio-Rad, Hercules, CA, USA). Quantification of Western blots relied on ImageJ software^9^.

**Tubuloids functional assay by fluorescein uptake**

Initially, tubuloids were removed from the surrounding BME by incubation with Dispase solution (20 min, at 37°C and 5% v/v CO_2_). The tubuloids were then dissociated in single cells by incubation with Accutase® solution (Invitrogen, Carlsbad, CA, USA) for 10 to 15 min at 37°C and 5% v/v CO_2_. The single cells derived from tubuloids were seeded in 6-well plates and maintained in expansion medium until 90% confluency. After the differentiation step of the respective experimental conditions (Figure 1a), the cell cultures were incubated with 1 μM fluorescein (Sigma-Aldrich, MO, USA) for 10 min at 37°C and 5% v/v CO_2._, in the presence or absence of 100 μM probenecid (Sigma-Aldrich, MO, USA), an inhibitor of OAT transport.^10^ The cell cultures were washed 3 times with ice-cold HBSS and were disrupted by incubating 100 μl 0.1 M NaOH for 10 min at 37°C. Fluorescence intensity was measured using GloMax® Explorer Multimode Microplate Reader (Promega, Madison, WI, USA), and expressed in arbitrary units (a.u.).

**Supplementary references**

1. Lindoso RS, Collino F, Bruno S, et al. Extracellular vesicles released from mesenchymal stromal cells modulate miRNA in renal tubular cells and inhibit ATP depletion injury. *Stem Cells Dev*. 2014;23.

2. Collino F, Lopes JA, Corrêa S, et al. Adipose-derived mesenchymal stromal cells under hypoxia: Changes in extracellular vesicles secretion and improvement of renal recovery after ischemic injury. *Cell Physiol Biochem*. 2019;52.

3. Cox J, Neuhauser N, Michalski A, Scheltema RA, Olsen J V., Mann M. Andromeda: A peptide search engine integrated into the MaxQuant environment. *J Proteome Res*. 2011;10:1794-1805.

4. Tyanova S, Temu T, Sinitcyn P, et al. The Perseus computational platform for comprehensive analysis of (prote)omics data. *Nat Methods*. 2016;13:731-740.

5. Perez-Riverol Y, Csordas A, Bai J, et al. The PRIDE database and related tools and resources in 2019: improving support for quantification data. *Nucleic Acids Res*. 2019;47(D1):D442-D450.

6. Pathan M, Keerthikumar S, Chisanga D, et al. A novel community driven software for functional enrichment analysis of extracellular vesicles data. *J Extracell vesicles*. 2017;6:1321455.

7. Koenderink JB, Geibel S, Grabsch E, De Pont JJHHM, Bamberg E, Friedrich T. Electrophysiological Analysis of the Mutated Na,K-ATPase Cation Binding Pocket. *J Biol Chem*. 2003;278:51213-51222.

8.Jansen J, Fedecostante M, Wilmer MJ, et al. Bioengineered kidney tubules efficiently excrete uremic toxins. *Sci Rep*. 2016;6:26715.

9. Schneider CA, Rasband WS, Eliceiri KW. NIH Image to ImageJ: 25 years of image analysis. *Nat Methods*. 2012;9:671-675.

10.Vriend J, Nieskens TTG, Vormann MK, et al. Screening of Drug-Transporter Interactions in a 3D Microfluidic Renal Proximal Tubule on a Chip. *AAPS J*. 2018;20:87.
