# Supplementary figures and images for: Differentiated kidney tubular cell-derived extracellular vesicles enhance maturation of tubuloids
Source: J Nanobiotechnology. 2022 Jul 15;20:326. doi: 10.1186/s12951-022-01506-6 (PMC9284832; doi:10.1186/s12951-022-01506-6)

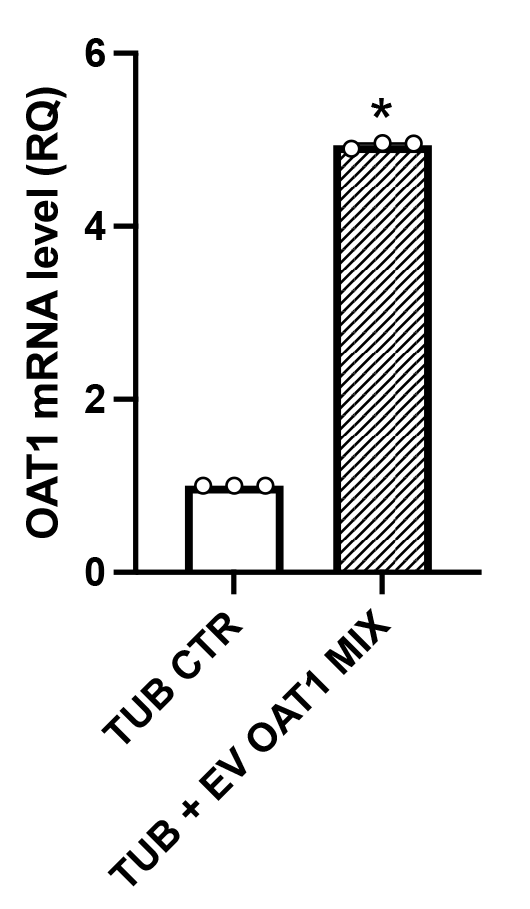

Supplement: Supplementary file 2 — Additional file 2: Figure S1. OAT1 mRNA levels within EV-OAT1 is compatible with the increased levels in tubuloids. The graph shows the total OAT1 mRNA levels present in EV-OAT1 and tubuloids that were previously isolated separately and then pooled to perform the qRT-PCR (TUB + EV-OAT1 MIX). The expression levels were compared to unstimulated tubuloids (TUB CTR). The data is expressed in relative quantification (RQ) in respect to the control condition (TUB CTR) (n = 3). Data represent mean ± SEM, *p < 0.05 with respect to TUB CTR group. [file 12951_2022_1506_MOESM2_ESM.tif]

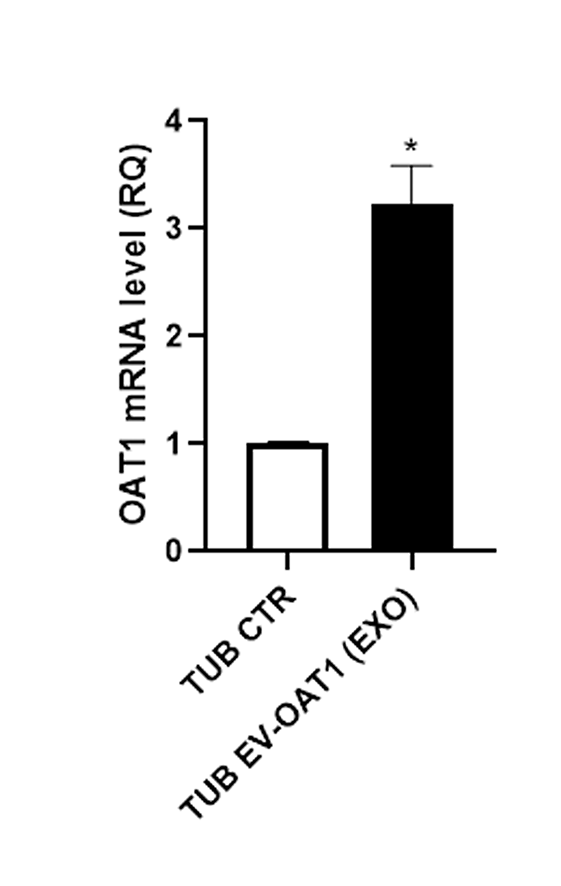

Supplement: Supplementary file 3 — Additional file 3: Figure S2. EV-OAT1 additionally purified from the CM maintained the upregulation of OAT1 in tubuloids. The graph shows the changes in the mRNA levels in the tubuloids culture under standard differentiation protocol (TUB CTR) and in the presence EV-OAT1 that were further isolated from the remaining medium using ExoQuick-TC. The data is expressed in relative quantification (RQ) in respect to the control condition (TUB CTR) (n = 3). Data represent mean ± SEM, *p < 0.05 with respect to TUB CTR group. [file 12951_2022_1506_MOESM3_ESM.tif]

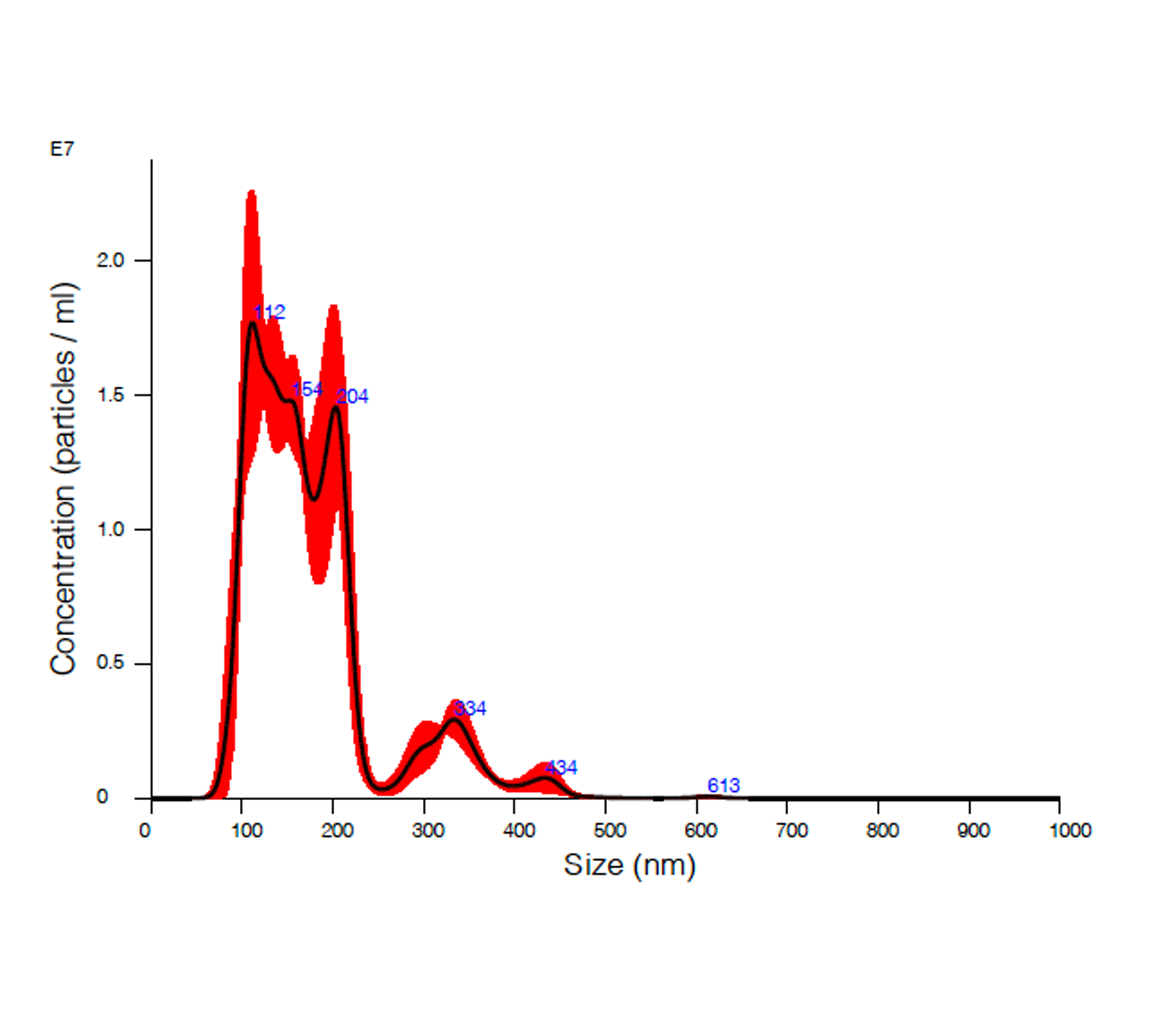

Supplement: Supplementary file 4 — Additional file 4: Figure S3. Nanoparticle Tracking Analysis representative graph of the EVs present in CM-OAT1, without isolation. The graph shows the size distribution of EVs (abscissa) and their concentration in particles/ml (ordinate). [file 12951_2022_1506_MOESM4_ESM.tif]
