# Supplementary material for: Differentiated kidney tubular cell-derived extracellular vesicles enhance maturation of tubuloids
Source: J Nanobiotechnology. 2022 Jul 15;20:326. doi: 10.1186/s12951-022-01506-6 (PMC9284832; doi:10.1186/s12951-022-01506-6)
Supplement: Supplementary file 5 — Additional file 5: Table S1. List of primers. [file 12951_2022_1506_MOESM5_ESM.docx]

| Primers | Forward | Reverse |
| --- | --- | --- |
| hATP1A1 | ACTTCCTCCGCATTTATGCTCATT | CTTAGCCTTGATGAACTTCA |
| hBCRP | CTTATGTTCCACGGGCCTGC | GGCTCTATGATCTCTGTGGCT |
| hHNF1A | CCCACCAAGCAGGTCTTCAC | AAGGTCTCGATGACGCTGTG |
| hHNF1B | AGAAGCGTGCCGCTCTGT | TGGTTGAATTGTCGGAGGATCT |
| hHNF4A | GGAATTTGAGAATGTGCAGGTGTTG | TGAGGTTGGTGCCTTCTGATG |
| hHPRT1 | ACATCTGGAGTCCTATTGACATCG | CCGCCCAAAGGGAACTGATAG |
| hMRP2 | GGTCATCCTTTACGGAGAACATCAG | AGGTCTGCCTCCGGACTGT |
| hMRP3 | CCAAGCACATCTTTGACCACG | CCTCTGCACCTTCCAACGC |
| hOAT1 | GGCACCTTGATTGGCTATGT | AAAAGGCGCAGAGACCAGTA |
| hOAT3 | CATCGGATCCAGACCCGGC | GGCCATGTTGAGGATCGGGA |
